# Human papillomavirus, Epstein–Barr virus, and *Candida albicans* co‐infection in oral leukoplakia with different degrees of dysplasia

**DOI:** 10.1002/cre2.435

**Published:** 2021-06-08

**Authors:** Alveiro T Erira, Andrea Fernanda Romo Navarro, Dabeiba Adriana García Robayo

**Affiliations:** ^1^ Facultad de Odontología Universidad Cooperativa de Colombia Bogotá Colombia; ^2^ Centro de Investigaciones Odontológicas ‐ Facultad de Odontología Pontificia Universidad Javeriana Bogotá Colombia

**Keywords:** leukoplakia, dysplasia, Epstein–Barr virus, Human papillomavirus, *Candida albicans*

## Abstract

**Objectives:**

To identify human papillomavirus (HPV), Epstein–Barr virus (EBV), and *Candida albicans* in oral leukoplakia with different degrees of dysplasia.

**Materials and methods:**

An observational, cross‐sectional, descriptive study was performed using 30 formalin‐fixed and paraffin‐embedded tissues from patients with clinical suspicion of leukoplakia and confirmed diagnosis of oral dysplasia. Histological analyses were performed by two pathologists (interobserver) and dysplasias were classified as mild, moderate, or severe. Conventional PCR was used to detect HPV and EBV viruses and *C. albicans*. To determine the association between each microorganism with different degrees of dysplasia a Chi‐square test was employed.

**Results:**

The tongue was the most common site for leukoplakias (71.4%) in females with a mean age of 50 years (ranging between 30 to 50 years old; 57.1%). EBV was the most frequently detected (73.3%), followed by HPV (43.3%), mainly of type 16 (40%), and *C. albicans* (23.3%). Significant differences were observed between degrees of dysplasia and HPV presence (*p* = 0.005). In lesions positive for HPV, EBV, and *C. albicans* the most frequent histological changes were hyperkeratosis, irregular interpapillary ridges, and loss of basal stratum cell polarity.

**Conclusion:**

Co‐infection with human papillomavirus, Epstein Barr virus, and *Candida albicans* in oral leukoplakia could be associated with dysplastic changes.

## 1 INTRODUCTION

Oral cancer affects any tissue in the oral cavity arising from a premalignant state, such as potentially malignant oral disorders (PMOD) to a malignant stage, such as oral squamous cell carcinomas (OSCC), as pointed out by the World Health Organization (Bansal et al., 2016; Ganesh et al., 2018). PMODs are classified as leukoplakia, erythroplakia, actinic cheilosis, oral submucosal fibrosis, among others (Warnakulasuriya et al., 2007). Oral leukoplakia has an estimated prevalence of 4.11%, where 1–18% of the cases progress to cancer (Escribano‐Bermejo & Bascones‐Martínez, 2009; Mello et al., 2018); Although erythroplakia is less prevalent than leukoplakia, with incidence rates between 0.02 and 0.83%, some of them have been described as microscopic dysplasias (Neville et al., 2018; Warnakulasuriya, 2018).

According to the Uppsala Symposium, Sweden 1994, clinical types of leukoplakia are classified as homogeneous and non‐homogeneous. Homogeneous leukoplakias are generally asymptomatic and are recognized by a uniform white lesion slightly elevated compared to surrounding mucosa with a thin appearance. The surface can often present fissures, wrinkles, or a corrugated surface in appearance. Non‐homogenous leukoplakias are defined as irregular red, nodular, or exophytic white superficial lesions and on occasion are symptomatic (Aguas & Lanfranchi‐Tizeira, 2004; Escribano‐Bermejo & Bascones‐Martínez, 2009; Martorell‐Calatayud et al., 2009).

Potential malignant lesions can also be classified as mild, moderate, or severe dysplasia (Martínez‐Sahuquillo Márquez et al., 2008; Warnakulasuriya & Ariyawardana, 2016).

Human papillomavirus (HPV) has been related to multiple hyperplastic, verrucous, and papillomatous lesions of the mucosa and skin epithelium (Petito et al., 2016). Several studies described HPV in 20% of oral leukoplakias, however their association has not been clearly established (Campisi et al., 2004; Martorell‐Calatayud et al., 2009; Perdomo‐Lara et al., 2020; Søland et al., 2020).

Epstein–Barr virus (EBV) has been implicated in multiple malignant neoplasms. Still, a causal association between leukoplakia and EBV is yet unclear (Braz‐Silva et al., 2008; Milagres et al., 2007). EBV replication increases in oral leukoplakias through mechanisms that favor its latency and persistence within the stratified squamous epithelium (Guidry et al., 2018).

*Candida* fungus is a common opportunistic pathogen in mucous membranes within the oral cavity and its activity increases in individuals with weakened immune systems (Sankari et al., 2015). *Candida albicans* is the most studied, representing a risk factor for premalignant transformation to malignant lesions in the oral cavity (Gainza‐Cirauqui et al., 2013; Verma et al., 2015). Furthermore, several mechanisms, such as nitrosamine and acetaldehyde formation have been associated with histopathological changes, such as epithelial hyperplasia, hyperkeratosis, micro‐abscesses, and chronic inflammation (Salvatori et al., 2016).

Because cancer is a progressive disease evolving from premalignant lesions to malignant stages; it is important to develop prognosis strategies during the early stages of the disease resulting from viral and fungal infections. These infections can result in tissue dysplasia associated with cancer advancement (Escribano‐Bermejo & Bascones‐Martínez, 2009; Pitiyage et al., 2009; Reibel, 2003).

Clinical and histological studies detecting HPV, EBV, and *Candida albicans* infections in premalignant lesions are necessary since their effect on malignant transformation is unclear. These associations could have a relevant clinical impact because these lesions can be reversed if diagnosed early and if infection receives a timely treatment.

## 2 MATERIALS AND METHODS

An observational cross‐sectional descriptive study was carried out with signed informed consent from all participants. The study was approved by the institutional ethics committee No. 014‐2015 in compliance with the Helsinki declaration protocols. A total of 45 samples were collected and analyzed; of these, only 30 met the selection criteria and were included for further analyses. Exclusion criteria included patients with oral cancer diagnosis, under antibiotic medication, and consuming alcohol. An oral biopsy was obtained from patients with presumptive oral leukoplakia diagnosis for histopathological dysplasia analysis.

### 2.1 Clinical and histological analysis of leukoplakias

After clinical examination, lesions were classified as homogeneous or non‐homogeneous. A biopsy was collected and divided into two parts; one half was used for diagnostic purposes. To this end, tissue specimens were formalin‐fixed, and paraffin embedded and stained with hematoxylin and eosin for histological analysis by two pathologists performing an inter‐observer and unbiased analysis. Leukoplakias were classified using the Smith and Pindborg's classification based on their degree of dysplasia as mild, moderate, or severe, (Krogh et al., 1987; Pindborg et al., 1997). Histological analyzes included: loss of polarity of the basal stratum cells, presence of more than one basal cell layer with basaloid appearance, increased nuclear/cytoplasmic ratio, drop‐shaped rete ridge, irregular epithelial stratification, increased number of mitotic figures with atypical cell shape, abnormally superficial mitoses, cellular and nuclear pleomorphisms, nuclear hyperchromatism, increased number and size of nucleoli, loss of cell cohesion, keratinization of isolated cells or in groups. If findings presented large discrepancies between both pathologists, then a third pathologist analyzed the slides and emitted his concept. This last evaluation was compared to the first two assessments, those with the highest concordance rate were selected (Kappa index >0.8).

Furthermore, the number of histological findings, cell atypia index and degree of dysplasia were graded as follows: Mild dysplasia: 11–25 points, moderate dysplasia: 26–45 points, and severe dysplasia: 46–75 points.

### 2.2 DNA extraction, quantification, and quality

The other half of the biopsy was used for DNA extraction using the Qiagen FFPE kit for paraffin‐embedded tissue sections, following the manufacturer's instructions. DNA was quantified and quality assessed using a Nanodrop T2000 and 3% agarose electrophoresis. DNA quality was verified by amplifying the beta‐globin gene (Table 1) by conventional PCR.

**TABLE 1:** Human papillomavirus, Human papillomavirus type 16, Epstein–Barr virus and *Candida albicans* primer sequences for studied microorganism

Target		5′ to 3′ primer sequence	Product size (bp)	Gene	References
Human papillomavirus	Forward	TTTGTTACTGTGGTAGATACTAC	150pb	L1	Kristoffersen et al., 2012
	Reverse	GAAAAATAAACTGTAAATCATATTC			
Human papillomavirus type 16	Forward	AGCAGAACCGGACAGAGCCCA	158pb.	E7	Erira et al., 2015
	Reverse	TCTGAGAACAGATGGGGCACACA			
Epstein–Barr virus	Forward	CTTAGAATGGTGGCCGGGCTGTAAAAT	209pb.	Ebna1	Jalouli et al., 2012
	Reverse	ATCCAGTACGTCTTTGTGGAGCCCAAG			
*Candida albicans*	Forward	CAGTTTTCGGTAAAGGGGT	180pb	ERG11	Own design
	Reverse	TGGCAACCCCATGAGTTTTT			
Beta‐globin	Forward	ACACAACTGTGTTCACTAGC	250pb.	β‐ Globin	Erira et al., 2015
	Reverse	GGAAAATAGACCAATAGGCAG			

### 2.3 HPV identification by conventional PCR

Specific primers were used to detect HPV in oral leukoplakias (Table 1), under the following PCR conditions: 1X GoTaq Buffer, 1 mM MgCl_2_, 1 μM GP5 +/GP6 + primers, 1.25 U GoTaq polymerase, 0.2 mM dNTPs, and 2 μl DNA sample (0.5 ng) in a total volume of 20 μl. Thermal cycling conditions were: 95° for 5 min, 95° for 1 min (1 cycle), 55° for 1 min, 72° for 1 min and 5 s (35 cycles), and one last cycle at 72° for 5 min. HeLa cells (positive for HPV) were used as a positive control and water as a negative control. Human papilloma virus PCR products were visualized in 3% w/v agarose gels in a Bio‐Rad Gel Doc XR+ documentation system. Each experiment was carried out in triplicate and internal controls were used.

### 2.4 Identification of HPV16 by conventional PCR

HPV‐positive samples were confirmed with specific primers for HPV16 (Table 1) under the following conditions: 1X GoTaq Buffer, 1 mM MgCl_2_, and 1 μM of primers, 1.25 U GoTaq polymerase, 0.2 mM dNTPs, and 2 μl (0.5 ng) DNA sample in a final volume 20 μl. Thermal cycling conditions were: 95° for 5 min, 95° for 1 min (1 cycle), 60° for 1 min, 72° for 1 min and 5 s (35 cycles), and the last cycle at 72° for 5 min. An HPV16 plasmid was used as a positive control (donated by Doctor Zur Hausen of the *Deutsches Krebsforschungszentrum*—DKFZ of Germany), and water was used as a negative control. PCR products for HPV 16 were visualized in 3% wt/vol agarose gel in a Bio‐Rad Gel Doc XR+ documentation system. Each experiment was carried out in triplicate and internal controls were used.

### 2.5 Identification of EBV by conventional PCR

EBV identification was carried out using specific primers for EBV (Table 1) under the following conditions: 1X GoTaq Buffer, 1 mM MgCl_2_, 0.1 μM primers, 1.25 U GoTaq polymerase, 0.2 mM dNTPs, 2 μl DNA sample (0.5 ng). Thermal cycling conditions were: 95° for 5 min, 95° for 1 min, 65° for 1 min (1 cycle), 72° for 1 min and 5 s (35 cycles), and the last cycle at 72° for 5 min. An EBV sequence was cloned in a pUC57 plasmid vector and used as a positive control, and water was used as a negative control. PCR products for EBV 16 were visualized in 3% wt/vol agarose gels in a Bio‐Rad Gel Doc XR+ documentation system. Each experiment was carried out in triplicate and internal controls were used.

### 2.6 Identification of *C. albicans* by conventional PCR

*C. albicans* was identified by specific primers (Table 1), under the following conditions: 1X GoTaq Buffer, 1 mM MgCl_2_, 1.25 U GoTaq Polymerase, 0.2 mM dNTPs, and 2 μl DNA samples (0.5 ng). The thermal cycling conditions were: 95° for 5 min, 95° for 1 min (1 cycle), 68° for 1 min, and 72° for 1 min and 5 s (35 cycles) and the last cycle at 72° for 5 min. These reactions were performed in a CFX96 Bio‐Rad device. *C. albicans* ATCC 90028 was used as a positive control, and water was used as a negative control. PCR products for *C. albicans* were visualized in 3% wt/vol agarose gels in a Bio‐Rad Gel Doc XR+ documentation system. Each experiment was carried out in triplicate and internal controls were used.

### 2.7 Statistical analysis

Data were analyzed using means, standard deviation, and table of frequencies to determine whether there was a relationship between HPV, EBV, and *C. albicans* and different grades of dysplasia. A Chi‐square test was performed using the SPSS statistical software, version 2016, with a significance of *p* < 0.05.

## 3 RESULTS

### 3.1 Characteristics of the population

The study cohort consisted mainly of females (78.5%); with age ranging between 30 to 50 years old (57.1%). A higher incidence was observed in urban areas (64.2%), where the tongue was the most common site for leukoplakias (21.4%; Table 2).

**TABLE 2:** Characteristics of the population

Gender	Female % (no)	Male % (no)
	78.5 (11)	21.4 (3)
Age (years)		
30–50	57.1 (8)	7.14 (1)
51–70	14.28 (2)	14.28 (2)
71–90	7.14 (1)	0 (0)
Origin		
Rural	14.28 (2)	7.14 (1)
Urban	64.2 (9)	14.28 (2)
Tobacco	7.14 (1)	0 (0)
Location of the lesion		
Tongue	71.4 (10)	21.4 (3)
Throat	7.14 (1)	0 (0)
Diagnosis		
Leukoplakia	78.5 (11)	21.4 (3)

### 3.2 Frequency of HPV, EBV, and *C. albicans* in oral leukoplakias according to clinical pattern and histological characteristics

Of the analyzed leukoplakias this study identified, 43.3% (*n* = 13) were positive for HPV (46% [*n* = 6] HPV16), 73.3% (*n* = 22) were positive for EBV, and 23.3% (*n* = 7) were positive for *C. albicans* (Table 3).

**TABLE 3:** Clinical Patterns and Frequency of infection by HPV, EBV, and *C. albicans* of leukoplakias with different grades of dysplasia

	Sample	HPV‐positive	HPV‐negative			EBV‐positive	EBV‐negative		*C. albicans‐*positive		*C. albicans‐*negative		
Characteristics	*n* (%)	*n* (%)	*n* (%)		*p*‐Value	*n* (%)	*n* (%)	*p*‐Value	*n* (%)		*n* (%)		*p*‐Value
Total	30 (100)	13 (43.3)	17 (56.6)			22 (73.3)	8 (26.6)		7 (23.3)		23 (76.6)		
Clinical pattern													
Homogeneous	16 (53.3)	6 (37.5)	10 (62.5)		0.78	10 (62.5)	6 (37.5)	0.35	3 (18.7)		13 (81.2)		0.81
Non‐homogeneous	14 (46.6)	7 (50)	7(50)			12 (85.7)	2 (14.2)		4 (28.5)		10 (71.4)		
Grade of dysplasia													
Mild	13 (43.3)	3 (23.0)	10(76.9)		0,005	8 (61.5)	5 (38.4)	0.44	1 (7.6)		12 (92.3)		0.016
Moderate	11 (36.6)	9 (81.8)	2 (18.1)			9 (81.8)	2 (18.1)		2 (18.1)		9 (81.8)		
Severe	6 (20)	1 (16.6)	5 (83.3)			5 (83.3)	1 (16.6)		4 (66.6)		2 (33.3)		

Out of the samples analyzed 53.3% of the leukoplakias were of the homogenous clinical type. In this group 37.5% were positive for HPV, 62.5% for EBV, and 18.7% for *C. albicans*. In contrast, 46.6% of leukoplakias of the non‐homogenous clinical type, 50% were positive for HPV, 85.7% for EBV, and 28.5% for *C. albicans*. (Table 3). However, no significant differences were observed between the clinical types and the presence of HPV, EBV, and *C. albicans* (Table 3).

Respecting histological characteristics, 43.3% had mild dysplasia, of which 23.0% were positive for HPV, 61.5% for EBV, and 7.6% for *C. albicans*. For the 36.6% with a moderate degree of dysplasia, 81.8% were positive for HPV and EBV, whereas 18.1% were positive for *C. albicans*. Last, for the 20% with a severe degree of dysplasia, 16.6% were positive for HPV, 83.3% for EBV and 66.6% for *C. albicans*. Interestingly, significant differences were observed between the degree of dysplasia and HPV and *C. albicans* with a *p* < 0.05 (Table 3).

### 3.3 Histological findings in leukoplakias positive for HPV, EBV, and *C. albicans*

**FIGURE 1:**
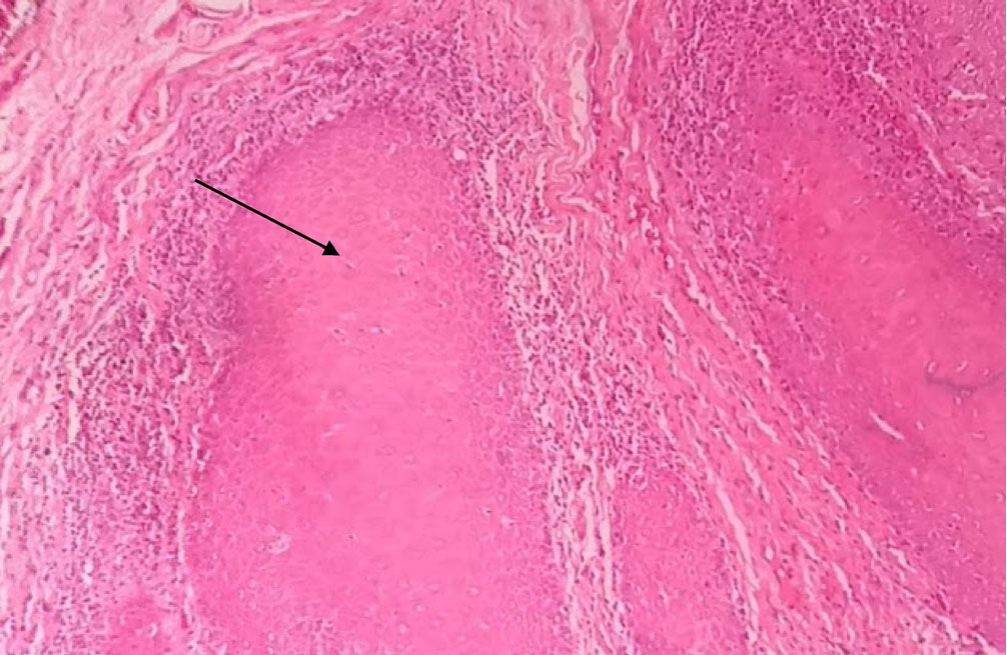
Irregular ridges (drop‐shaped rete ridges) H&E 10×, severe dysplasia

**FIGURE 2:**
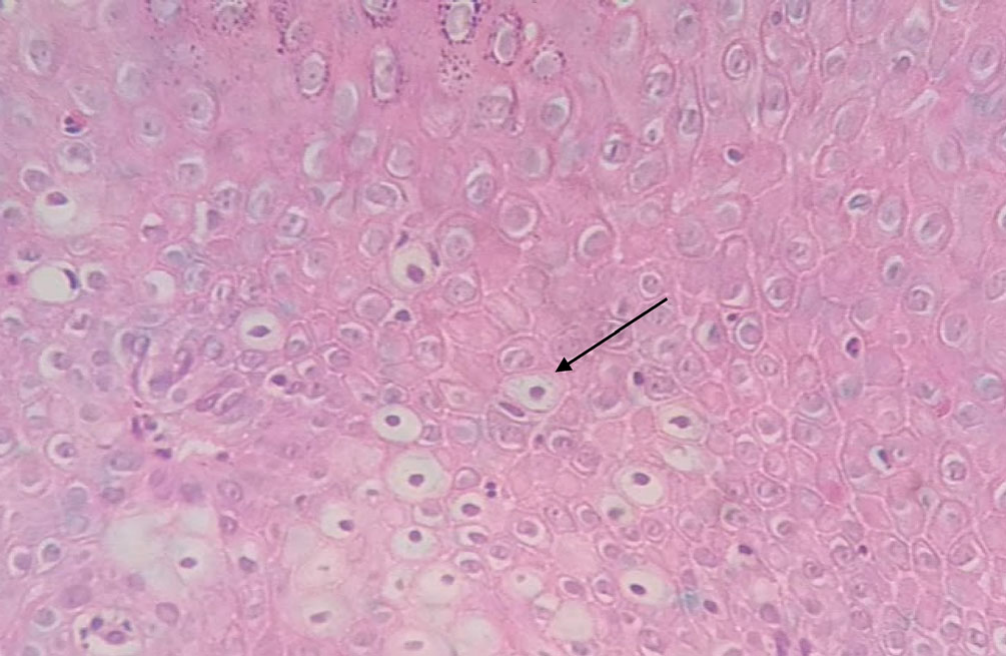
Cells with koilocytic appearance. H&E 40×, moderate grade dysplasia

**FIGURE 3:**
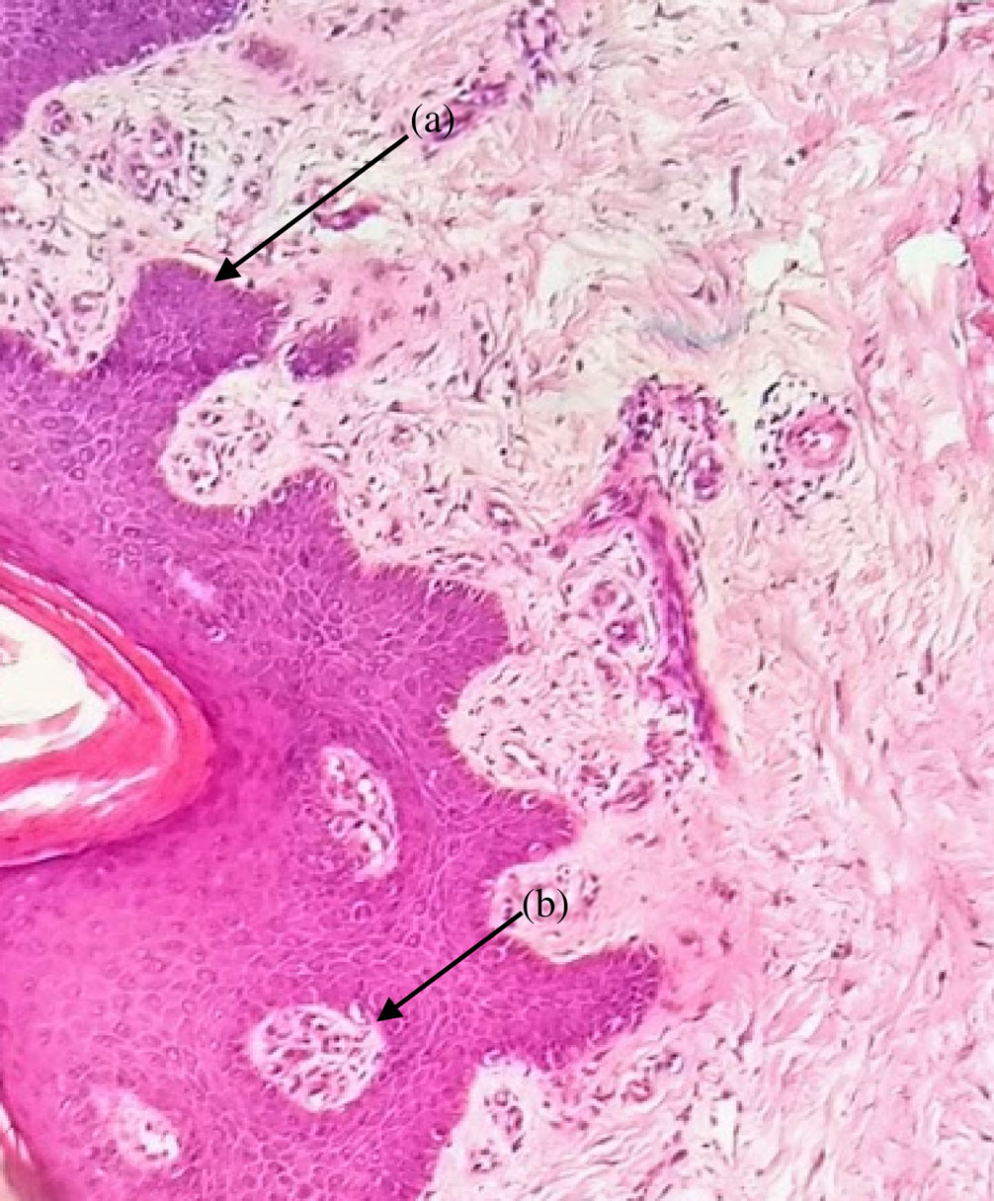
(a) Severe loss of epithelial adhesion and islets of connective tissue in the epithelium; (b) keratin pearls within rete pegs. H&E 10×, moderate grade dysplasia

Histological abnormalities such as hyperkeratosis, irregular ridges, drop‐shaped rete ridges, loss of polarity of basal cells, more than one basal cell hyperplasia, severe loss of epithelial adhesion, islets of connective tissue in the epithelium, cells with koilocytic appearance were commonly identified in mild grade of dysplasia positive for HPV, EBV, and *C. albicans*. (Table 4 and Figure 1, 2, 3).

**TABLE 4:** Histological characteristics of positive leukoplakias for HPV, EBV, and *C. albicans*

	Mild %			Moderate %			Severe %			
Dysplastic changes	VPH+	VEB+	*C. albicans*+	VPH+	VEB+	***C. albicans+***	VPH+	VEB+	*C. albicans*+
Hyperkeratosis	14.29	26.32	12.50	35.71	26.32	25.00	7.14	10.53	37.50
Irregular ridges ‐ Drop‐shaped papillae	0.00	15.79	12.50	35.71	26.32	25.00	14.29	5.26	25.00
Loss of basal stratum cells polarity	7.14	5.26	0.00	35.71	26.32	12.50	7.14	10.53	37.50
More than one basal cell layer with basaloid appearance	7.14	5.26	0.00	42.86	31.58	25.00	7.14	10.53	37.50
Increase in cytoplasm‐nuclei ratio	0.00	0.00	0.00	14.29	10.53	0.00	7.14	5.26	12.50
Atypical mitoses	0.00	0.00	0.00	0.00	0.00	0.00	7.14	5.26	37.50
Mitoses in the upper half of the epithelium	0.00	0.00	0.00	0.00	0.00	0.00	7.14	5.26	25.00
Cellular and nuclear pleomorphism	0.00	0.00	0.00	57.14	42.11	25.00	14.29	15.79	50.00
Nuclear hyperchromatism	0.00	0.00	0.00	21.43	15.79	12.50	7.14	10.53	50.00
Severe loss of epithelial adhesion	14.29	10.53	0.00	28.57	21.05	0.00	0.00	5.26	25.00
Islets of connective tissue in the epithelium	7.14	5.26	0.00	0.00	0.00	12.50	7.14	10.53	25.00
Keratin pearl in deep stratum	0.00	0.00	0.00	7.14	5.26	0.00	0.00	5.26	25.00
Keratinized cells with pyknotic nucleus	0.00	0.00	0.00	7.14	5.26	12.50	7.14	5.26	25.00
Acanthosis	0	0.00	0.00	28.57	21.05	25.00	14.29	15.79	37.50
Cells with koilocytic appearance	7.14	10.53	0.00	28.57	21.05	12.50	14.29	10.53	12.50

**FIGURE 4:**
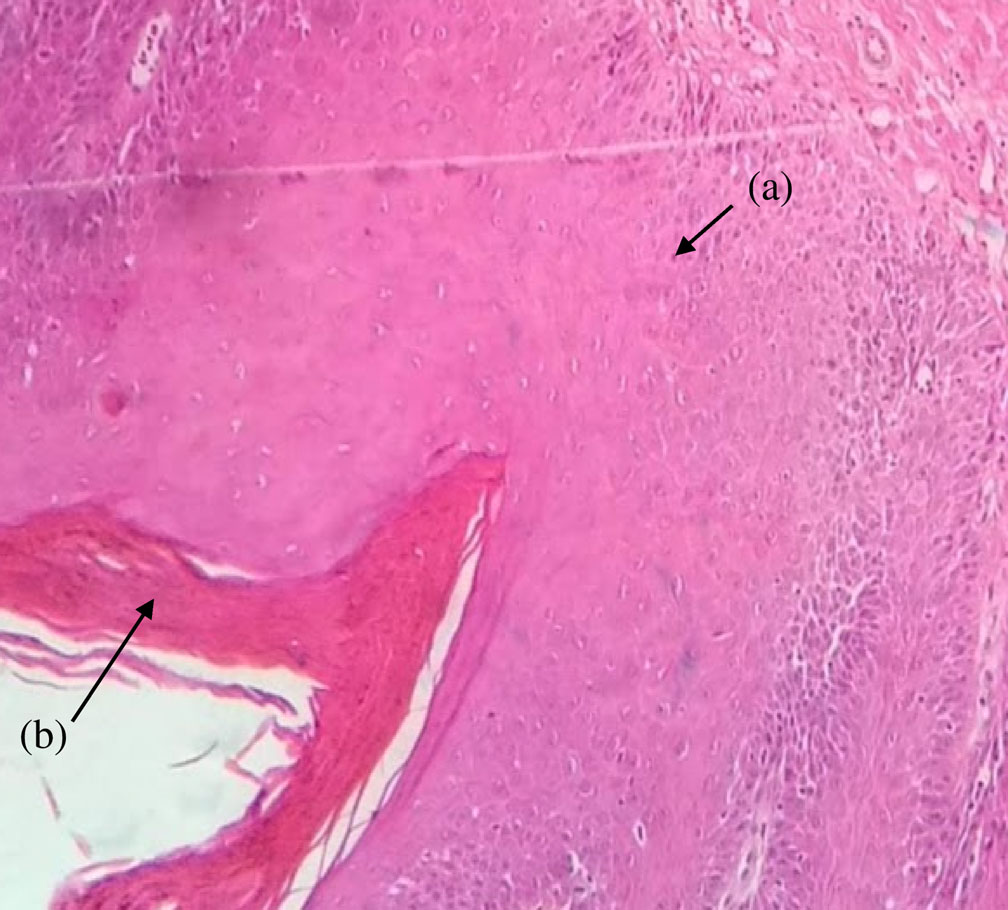
(a) Acanthosis; (b) hyperkeratosis. H&E 10×, moderate grade dysplasia

**FIGURE 5:**
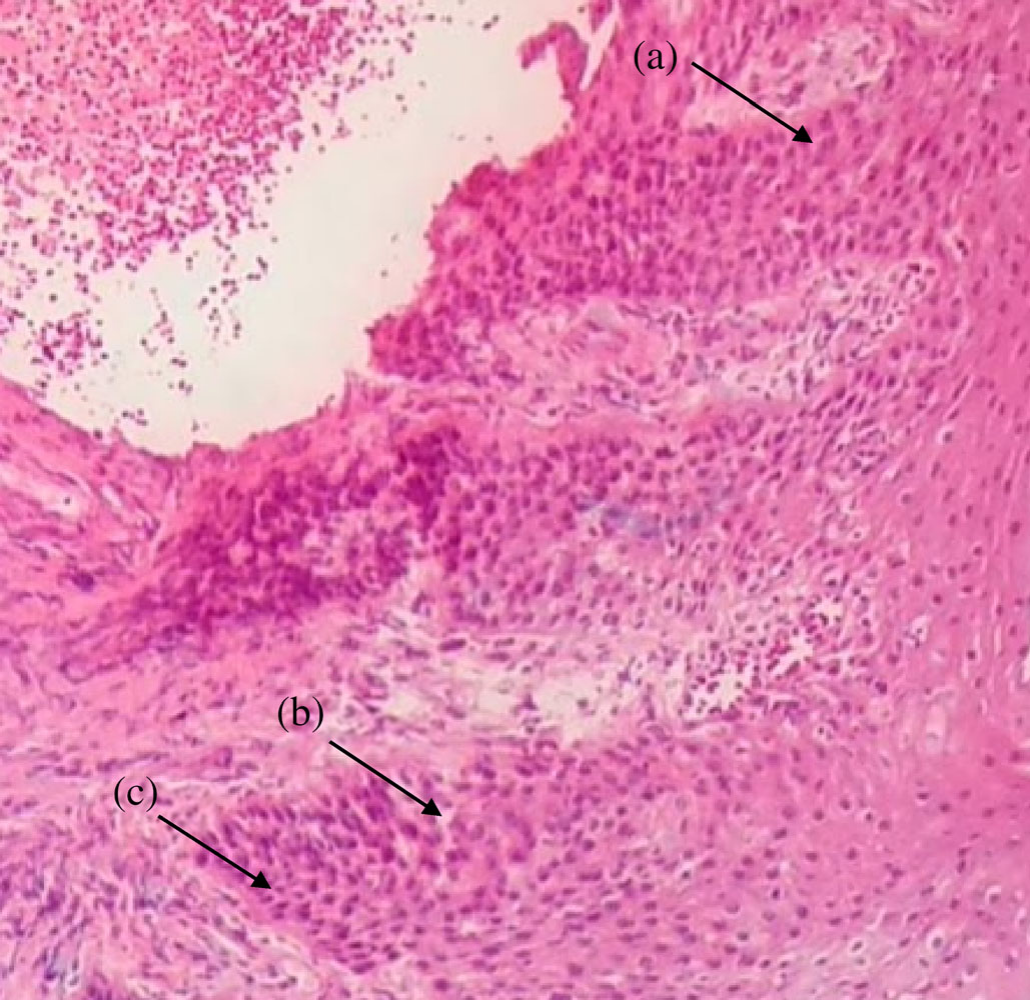
(a) Cellular and nuclear pleomorphism. (b) Loss of polarity of basal cells. (c) Nuclear hyperchromatism. H&E 10×, moderate grade dysplasia

In HPV, EBV, and *C. albicans‐*positive samples moderate grade of dysplasia were characterized. Frequently observed histological abnormalities were hyperkeratosis, irregular interpapillary ridges, loss of polarity of basal cells, presence of more than one layer of basal cells with basaloid appearance, increased nuclear‐cytoplasmic ratio, cellular and nuclear pleomorphism, nuclear hyperchromatism, severe loss of epithelial adhesion, keratin pearls within rete pegs, pyknotic nuclei keratinized cells, acanthosis and cells with koilocytic appearance (Table 4 and Figure 3, 4, 5).

Severe grade of dysplasia and positive for HPV, EBV, and *C. albicans* displayed hyperkeratosis, irregular interpapillary ridges, loss of basal stratum cell polarity, presence of more than one layer of basal cells with basaloid appearance, nuclear‐cytoplasmic ratio increase, increased number of mitotic figures, abnormally superficial mitosis, cellular and nuclear pleomorphism, nuclear hyperchromatism, severe loss of epithelial adhesion, islets of connective tissue in the epithelium, keratin pearls within rete, pyknotic nuclei keratinized cells, acanthosis and cells with koilocytic appearance (Table 4 and Figures 1, 2, 3, 4, 5).

## 4 DISCUSSION

Dysplastic lesions have been defined by various categorical systems to standardize their terminology over the years. In 1969, Smith‐Pindborg determined the rate of cellular atypia based on cellular microscopic changes. The level of dysplasia was defined on a grading system, where from 0 to 10 points represents lack of dysplasia, from 11 to 25 points corresponds to a mild dysplasia, from 26 to 45 points defines a moderate dysplasia and from 46 to 75 points portrays a severe dysplasia (Ranganathan & Kavitha, 2019). Due to the subjective character when performing a histological evaluation, the authors attempted to describe the diagnosis of epithelial dysplasia with a certain objective and quantitative character according to the established parameters and available resources (Lingen et al., 2017).

Subsequently, the WHO (2005) introduced the term “ epithelial precursor lesions” defined as epithelium alterations with a greater probability of progression to squamous cell carcinoma (Reibel et al., 2017), based on observed cellular changes and their epithelial location. Presence and severity of cellular atypia and the architectural features helped define dysplasia's diagnostic criteria, which were basically the same as those previously defined by Pindborg under another nomenclature. In 2017, the WHO published a revision of the 2005 classification system, with changes such as elimination of squamous hyperplasia and carcinoma in situ, which is considered a synonym for severe dysplasia. Never the less, the overall perception was a return to the initial 1978 WHO classification, where only mild, moderate and severe dysplasia were taken into account (Reibel et al., 2017). Although several classifications of dysplasia have been proposed through the years, they all show a certain degree of limitation regarding subjectivity in determining changes and variability in inter‐observer results. Collectively, what becomes relevant is the identification of epithelial cell changes in dysplastic lesions to recognize their potential malignancy (Koch et al., 2011).

Therefore, in the present study the Pindborg classification was used (Krogh et al., 1987; Pindborg et al., 1997) taking into account the 13 criteria for classifying mild, moderate and severe dysplasia, determining the severity of the dysplasia, and the association with viral and fungal infections.

In the study of pre‐malignant and malignant lesions presence of infectious agents in the oral cavity has gained importance because some lesions develop in the absence of the main risk factors, such as alcohol and tobacco consumption. Moreover, certain pathogenic microorganisms such as HPV (Nielsen et al., 1996), EBV (Guidry et al., 2018) and *Candida albicans* (Chiu et al., 2011; Verma et al., 2015) can alter cellular function and induce histological alterations in oral cavity tissues.

Among the most prevalent oral cavity disorders, oral leukoplakia has a 4.11% malignancy potential mainly affecting the tongue (Mangold et al., 2016). In fact, in the present study the anatomical region most frequently affected was the tongue (21.4%). In agreement with these findings Nagao et al. and Yang et al. have also described leukoplakia lesions in the tongue (Nagao et al., 2015; Yang et al., 2019). Furthermore, leukoplakias have been described in tongue and the floor of the mouth (Brouns et al., 2013; Kuriakose et al., 2016), tongue and gingiva (Kuribayashi et al., 2012), dorso‐laterally side of the tongue and buccal mucosa (Seijas‐Naya et al., 2012). In contrast, different findings were reported in others parts of the oral cavity, like the gingiva (Yang et al., 2009), gingival groove (Nagao et al., 2015), groove, cheeks, and lips (Pietruska et al., 2014), gums, mucous of the cheeks and palate (López‐Jornet & Camacho‐Alonso, 2013), buccal mucosa, mandibular mucosa and lips (Kawczyk‐Krupka et al., 2012).

In regard to gender, 78.5% of the women presented oral leukoplakia, similar to what was reported by Kristoffersen et al., 2012, who studied 50 samples of leukoplakia and found females were the most frequently affected (44%; Kristoffersen et al., 2012). Similar findings were reported by Szarka et al., who conducted a study in 2010 with 44 leukoplakia samples, and observed the study population with the highest incidence were females (30 cases; Szarka et al., 2009). Conversely, in 2009 Yang et al. reported that male patients presented a higher incidence (134/167 cases; Yang et al., 2009).

Concerning age, herein we established the average age was 50 years old (range, 30–70 years), similar to that reported in 2009 by Yang et al. who described a mean age of 48 years old (<65 years old) in patients with oral leukoplakia (Yang et al., 2009). In 2012, Kristoffersen reported an average age of 56 years (Kristoffersen et al., 2012) and, Szarka et al. (2009) reported an average age of 56 years (Szarka et al., 2009).

The aforementioned data shows that although oral disorders with malignant potentials, such as leukoplakia, are diagnosed more frequently in advanced age; timely diagnosis is challenging, due to lack of clinical examination follow‐up and absence of symptoms (Warnakulasuriya, 2018). Regardless, based on evidence it has been demonstrated the natural history of the disease is progressive and preventable (Goodson et al., 2015).

In the present study, the homogenous clinical type was the most frequent (55.71%). However, others studies have described these clinical type in other sites of the oral cavity, such as gums (56%), palate (9%), lips (16.6%), tongue (25.7%), and the floor of the mouth (19.8%; Brouns et al., 2013; Kuriakose et al., 2016; López‐Jornet & Camacho‐Alonso, 2013; Nagao et al., 2015; Pietruska et al., 2014; Yudenia & Cabarcos, 2018). Epstein–Barr virus was most frequently (85.7%) observed in the non‐homogeneous clinical type and could influence malignant changes in a given period.

We determined that leukoplakias were most frequently infected by EBV (73.3%), followed by HPV (43.3%), in addition to *C. albicans* infection (23.3%). The main HPV serotype was 16, which was found in 40% of the samples included. Presence of these infections could lead to dysplasia in oral cavity tissues and induce malignant processes (Stojanov & Woo, 2015).

Human papillomavirus infection is an important risk factor for oral leukoplakia and has been associated with carcinogenic processes (Angiero et al., 2010; Chen & Zhao, 2017). Since viruses are obligate intracellular microorganisms, they can induce cellular alterations, which can result in dysplasias (Lerman & Woo, 2014; Stojanov & Woo, 2015). This was evidenced in this study, where a significant difference (*p* = 0.005) was observed between presence of HPV and cellular atypia at the level of dysplasia (mild, moderate, and severe; Table 2). According to our results, the most frequent histological changes were hyperkeratosis, irregular ridges, teardrop‐shaped papillae, more than one basal cell layer with basaloid appearance, cellular and nuclear pleomorphism, and cells with koilocytic appearance in HPV‐infected moderate‐grade leukoplakia.

Human papillomavirus infection in oral cavity tissues is quite frequent and has been reported in healthy individuals (Esquenazi et al., 2010; Kristoffersen et al., 2012), as well as patients with leukoplakia (Angiero et al., 2010; Kristoffersen et al., 2012; Szarka et al., 2009). Human papillomavirus‐16 has been mainly identified in premalignant lesions (Bhosale et al., 2016; Saghravanian et al., 2011). However, there is still controversy regarding the association between HPV and cancer (Kristoffersen et al., 2012; Yang et al., 2009), because it is not always present.

Some HPV mechanisms that could cause malignant transformation include *E6* and *E7* expression, two oncoproteins that interfere with p53 and pRB tumor suppressor protein activity within the cell cycle, thus promoting uncontrolled proliferation and, subsequent cancer (Erira et al., 2015; Rampias et al., 2009). These mechanisms could also be related to dysplastic changes at early stages (Chen & Zhao, 2017; Khanal et al., 2017).

Furthermore, EBV infection has also been frequently reported in healthy individuals in addition to leukoplakia (Kikuchi et al., 2016; Sand et al., 2002). Even though it is an important risk factor for malignant transformation (Acharya et al., 2015), the association between infection, premalignant lesion onset, and cancer is still controversial (Kikuchi et al., 2017; Søland et al., 2020). Several mechanisms responsible for these changes could be based on *LMP1* protein expression, which allows for viral replication within cells and could alter cell function and induce dysplastic changes (Ali et al., 2015; Kikuchi et al., 2016). The findings of the present study include several histological changes, such as cellular and nuclear pleomorphism, more than one layer of basal cells with basaloid appearance, and hyperkeratosis in leukoplakia positive for EBV.

Co‐infection with HPV and EBV has been reported in pre‐cancerous lesions and OSCC. (Jalouli et al., 2010, 2012; Jiang et al., 2015; Sharma et al., 2019). This viral interaction has allowed us to evidence that HPV infection is important, however, not sufficient, for cancer development, since not all individuals infected with HPV progress into cancer (Guidry & Scott, 2017). However, presence of other microorganisms, such as EBV and *C. albicans*, may trigger a synergistic mechanism or enhance HPV's malignancy.

*Candida albicans* oral cavity infection is highly frequent (Gainza‐Cirauqui et al., 2013; Sankari et al., 2015; Vuèkoviæ & Bokor‐bratiæ, 2004) and has been reported as an key risk factor, and frequently identified in oral leukoplakia (Bakri et al., 2014; Gainza‐Cirauqui et al., 2013; Sankari et al., 2015). Although it is considered a public health issue (Dilhari et al., 2016), the association between infection and leukoplakias is still unresolved.

It is important to highlight that this fungus can invade superficial epithelial layers, and most strains perform what is known as “commutation,” which allows the fungus to change phenotypes with different morphological and functional properties (Sitheeque & Samaranayake, 2003). This, in turn, could induce histological changes, such as those reported in the present investigation, which has also been reported in other studies (Gujral et al., 2010).

This fungus increases its activity in humans with certain immune deficiencies (Salvatori et al., 2016). Moreover, within the oral cavity it is a risk factor for premalignant lesion transformation into malignant lesions (Gainza‐Cirauqui et al., 2013; Verma et al., 2015), mainly in the presence of tissues injured by trauma, maceration, atrophy or friction. Some mechanisms, such nitrosamine and acetaldehyde production have been associated with histopathological changes, including epithelial hyperplasia, hyperkeratosis, microabscesses, and chronic inflammation (Salvatori et al., 2016). However, this study identified the most frequent histological changes were loss of basal stratum cell polarity, atypical mitoses, nuclear hyperchromatism, and acanthosis in severe grade leukoplakias positive for *C. albicans*.

## 5 CONCLUSION

Leukoplakia on the tongue was most frequently infected by EBV, and the presence of HPV and *C. albicans* could be associated with dysplastic disorders.

## AUTHOR CONTRIBUTIONS

**Alveiro. T. Erira:** Conceptualization, Methodology, Validation, Formal análisis, Investigation, Resources, Writing ‐ Original Draft, Writing ‐ Review & Editing, Visualization, Supervision, Project administration, Funding acquisition. **Andrea F. R. Navarro:** Methodology, Validation, Formal análisis, Writing ‐ Original Draft, Writing ‐ Review & Editing. **Dabeiba A. G. Robayo:** Conceptualization, Methodology, Writing ‐ Original Draft, Writing ‐ Review & Editing.

## CONFLICT OF INTEREST

The authors declare there is no conflict of interest.

## ETHICS STATEMENT

The study was approved by the Ethics committee of the Universidad Cooperativa de Colombia—Bogotá Colombia (No. 014‐2015). All samples were collected from participants after signed informed consent under the ethical norms of the declaration of Helsinki.

## Data Availability

The data is not available because it compromises ethical standards.
